# Pseudomonas aeruginosa Aggregate Formation in an Alginate Bead Model System Exhibits *In Vivo*-Like Characteristics

**DOI:** 10.1128/AEM.00113-17

**Published:** 2017-04-17

**Authors:** Majken Sønderholm, Kasper Nørskov Kragh, Klaus Koren, Tim Holm Jakobsen, Sophie E. Darch, Maria Alhede, Peter Østrup Jensen, Marvin Whiteley, Michael Kühl, Thomas Bjarnsholt

**Affiliations:** a Costerton Biofilm Centre, Department of Immunology and Microbiology, Faculty of Health and Medical Sciences, University of Copenhagen, Copenhagen, Denmark; b Marine Biology Section, Department of Biology, University of Copenhagen, Elsinore, Denmark; c College of Natural Sciences, The University of Texas at Austin, Austin, Texas, USA; d Department of Clinical Microbiology, Copenhagen University Hospital, Copenhagen, Denmark; e Climate Change Cluster, University of Technology Sydney, Ultimo, New South Wales, Australia; University of Bayreuth

**Keywords:** Pseudomonas aeruginosa, biofilm, spatial structure, chronic infection, antibiotics, growth, respiration, model system

## Abstract

Alginate beads represent a simple and highly reproducible *in vitro* model system for diffusion-limited bacterial growth. In this study, alginate beads were inoculated with Pseudomonas aeruginosa and followed for up to 72 h. Confocal microscopy revealed that P. aeruginosa formed dense clusters similar in size to *in vivo* aggregates observed *ex vivo* in cystic fibrosis lungs and chronic wounds. Bacterial aggregates primarily grew in the bead periphery and decreased in size and abundance toward the center of the bead. Microsensor measurements showed that the O_2_ concentration decreased rapidly and reached anoxia ∼100 μm below the alginate bead surface. This gradient was relieved in beads supplemented with NO_3_^−^ as an alternative electron acceptor allowing for deeper growth into the beads. A comparison of gene expression profiles between planktonic and alginate-encapsulated P. aeruginosa confirmed that the bacteria experienced hypoxic and anoxic growth conditions. Furthermore, alginate-encapsulated P. aeruginosa exhibited a lower respiration rate than the planktonic counterpart and showed a high tolerance toward antibiotics. The inoculation and growth of P. aeruginosa in alginate beads represent a simple and flexible *in vivo*-like biofilm model system, wherein bacterial growth exhibits central features of *in vivo* biofilms. This was observed by the formation of small cell aggregates in a secondary matrix with O_2_-limited growth, which was alleviated by the addition of NO_3_^−^ as an alternative electron acceptor, and by reduced respiration rates, as well as an enhanced tolerance to antibiotic treatment.

**IMPORTANCE** Pseudomonas aeruginosa has been studied intensively for decades due to its involvement in chronic infections, such as cystic fibrosis and chronic wounds, where it forms biofilms. Much research has been dedicated to biofilm formation on surfaces; however, in chronic infections, most biofilms form small aggregates of cells not attached to a surface, but embedded in host material. In this study, bacteria were encapsulated in small alginate beads and formed aggregates similar to what is observed in chronic bacterial infections. Our findings show that aggregates are exposed to steep oxygen gradients, with zones of oxygen depletion, and that nitrate may serve as an alternative to oxygen, enabling growth in oxygen-depleted zones. This is important, as slow growth under low-oxygen conditions may render the bacteria tolerant toward antibiotics. This model provides an alternative to surface biofilm models and adds to the comprehension that biofilms do not depend on a surface for formation.

## INTRODUCTION

Bacteria associated with humans, both in health and in disease, are predominantly organized in aggregated cell consortia, also known as biofilms. Biofilm aggregates are characteristic of chronic bacterial infections but their *in vivo* function, metabolism, and growth remain largely unknown due to the lack of suitable *in vitro* models (1, 2). In this study, we employed the opportunistic pathogen Pseudomonas aeruginosa, which plays a major role in chronic infections and is a key model organism for studying biofilm formation and persistence in chronic infections. P. aeruginosa is capable of causing acute and chronic infections in wounds (3) and in the lungs of cystic fibrosis (CF) patients (4, 5). The ability to persist in chronic infections is ascribed to the biofilm-forming capability of P. aeruginosa, which enables it to survive antibiotic treatment and evade host defenses (6, 7). This persistence is governed by a high adaptability of P. aeruginosa to environmental changes thought to be a result of its highly flexible metabolism (8).

Most knowledge on medically relevant bacterial biofilms is based on the application of *in vitro* continuous-flow cell systems and 96-well plates, where biofilms are grown on surfaces and form a variety of structures, including the characteristic mushroom structure (9), albeit these structures have never been observed *in vivo* (1). Nevertheless, such surface-associated biofilms can exhibit *in vivo*-like characteristics with regard to diffusion-limited bacterial growth and differential tolerance across the biofilm depth to antibiotics and host immune responses (10). While this *in vitro* model may be well suited for studying some infections, including urinary tract or catheter biofilm formation, the opportunistic pathogen P. aeruginosa has never been observed in biofilms colonizing the epithelial cell surface in CF airways or in chronic wounds (7, 11). Instead, these chronic infections are characterized by the presence of nonattached relatively small (<50- to 100-μm wide) cell aggregates embedded in host material, such as wound bed slough or CF lung mucus constituting what we now term the secondary matrix. In the secondary matrix, bacterial aggregates are often surrounded by dense aggregations of host immune cells, such as polymorphonuclear leukocytes (PMNs), which contribute to a chronic state of inflammation (12, 13). The intense O_2_ consumption by the respiratory burst of activated PMNs (14) facilitates strong local O_2_ depletion (15), which may render bacterial cell aggregates surrounded by PMNs largely anoxic (16, 17).

Under low-O_2_ conditions in biofilm cell aggregates and in endobronchial secretions in CF airways (18), P. aeruginosa can grow anaerobically by utilizing the alternative electron acceptors, NO_3_^−^ and NO_2_^−^, which are present in appropriate amounts (19, 20). In chronic wounds, P. aeruginosa is observed colonizing deep wound regions, 50 to 60 μm from the wound surface (21), which may also be attributed to its capability for anaerobic respiration.

The physiochemical conditions, such as hypoxia and anoxia, and the embedment of aggregates in a secondary matrix are hard to mimic in present *in vitro* model systems. The O_2_ status of biofilm aggregates is thought to have a strong impact on the antibiotic tolerance of pathogenic bacteria (22). Low growth rates under hypoxia or anoxia in biofilms associated with chronic infections can have serious implications, as antibacterial treatment strategies are usually developed for aerobic fast-growing planktonic bacteria but have little impact on biofilm infections (23). To study the persistence of pathogenic bacteria such as P. aeruginosa in chronic infections, there is a need for better *in vitro* biofilm models mimicking the central traits of the *in vivo* biofilm. This would enable us to gain new knowledge of the central aspect of biofilm infections, as well as to improve diagnostics and testing of new strategies for antimicrobial treatment under *in vivo*-like conditions. In this study, we demonstrate a simple, reproducible *in vitro* biofilm system enabling P. aeruginosa to grow as spatially structured aggregates with size and growth characteristics similar to those seen in CF lungs (17) and chronic wounds (1).

## RESULTS

### Bacterial growth and organization in alginate beads.

**FIG 1:**
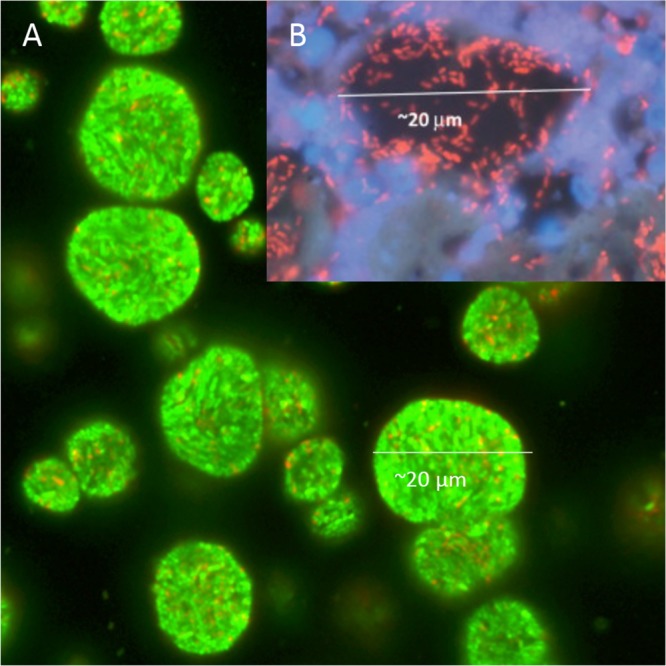
*In vitro* and *in vivo* aggregates of P. aerugionsa. (A) Confocal laser scanning microscopy (CLSM) image of alginate-encapsulated green fluorescent protein (GFP)-tagged P. aeruginosa PAO1 (green) grown *in vitro* for 24 h. (B) CLSM image of *in vivo* aggregate of P. aeruginosa (red) from chronic infected cystic fibrosis (CF) lung visualized with a peptide nucleic acid (PNA) fluorescence *in situ* hybridization (FISH) probe. The polymorphonuclear leukocytes surrounding the aggregate are stained with DAPI (4′,6-diamidino-2-phenylindole; blue). Reprinted from Bjarnsholt et al. (1) with permission.

**FIG 2:**
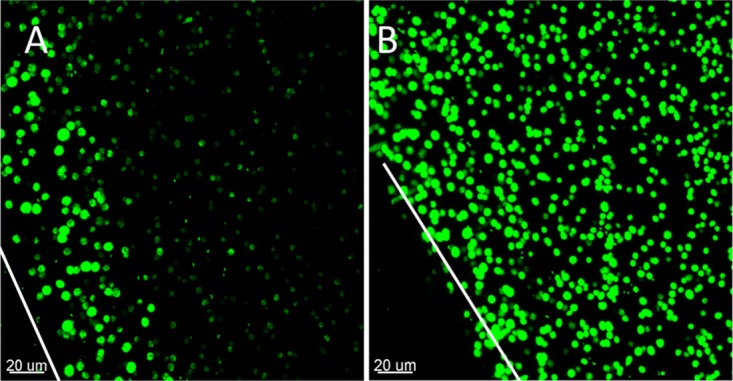
Alginate-encapsulated GFP-tagged P. aeruginosa PAO1 after 24 h of growth. CLSM images are of controls without (A) and those with (B) NO_3_^−^. The white lines correspond to the edge of the alginate beads (z lines), which are cut in half and imaged from the cut surface.

**FIG 3:**
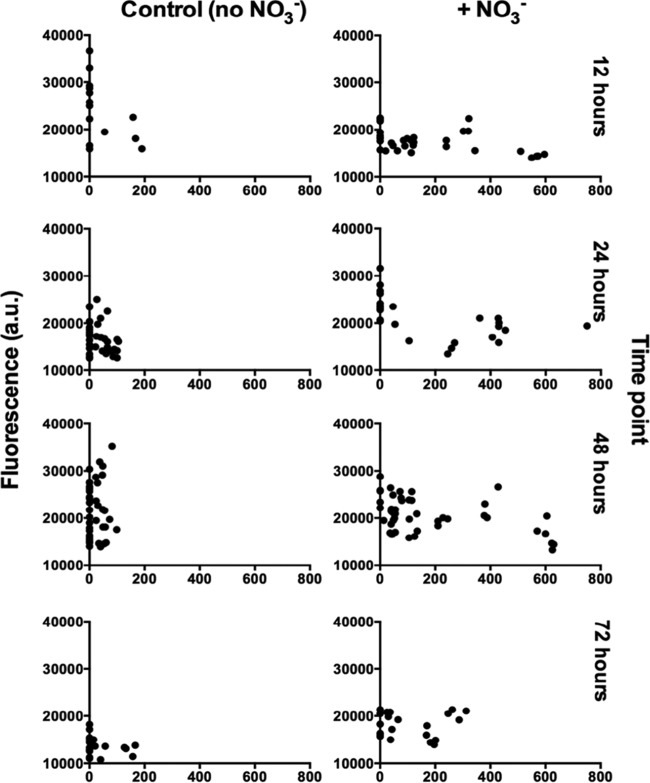
Growth rates expressed as relative fluorescence in arbitrary units (a.u.) as a function of depth within the alginate beads, grown with and without NO_3_^−^ and at different growth stages. P. aeruginosa was fluorescently labeled with a Texas Red-conjugated PNA-FISH probe and imaged by CLSM, and the fluorescence intensities were quantified in ImageJ.

When grown in alginate beads, P. aeruginosa formed micrometer-sized (∼100 to 200 μm^3^) heterogeneously distributed dense aggregates similar to those observed in the CF lung (Fig. 1). The aggregates formed primarily at the periphery of the beads, but this tendency was alleviated by the addition of the alternative electron acceptor NO_3_^−^ (Fig. 2). We found that P. aeruginosa colonies grew deeper into the alginate beads when supplemented with NO_3_^−^ (mean depth, 155.1 μm with NO_3_^−^ versus 33.6 μm without NO_3_^−^, *P* < 0.001) (Fig. 3). The apparent growth rates of alginate-encapsulated P. aeruginosa were estimated by quantitative peptide nucleic acid fluorescence *in situ* hybridization (PNA-FISH), based on a previously described linear correlation between relative fluorescence of PNA-FISH-stained rRNA molecules in P. aeruginosa and the growth rate (17). We found no significant correlation between growth depth and apparent growth rate in the presence of NO_3_^−^. By contrast, we found a significant negative correlation between growth depth and apparent growth rate without added NO_3_^−^ (*P* = 0.040). Interestingly, P. aeruginosa growing without NO_3_^−^ showed a higher growth rate during the initial 12 h of growth than with NO_3_^−^ (*P* < 0.001). After 24 h, the apparent growth rate was highest for the NO_3_^−^ group (*P* < 0.001), while there was no significant difference between the two groups after 48 h, and after 72 h, the apparent growth rate was again the highest for the NO_3_^−^ group (*P* < 0.001). There was a negative correlation between time and apparent growth rate for beads without NO_3_^−^ (*P* = 0.005) but not for beads with NO_3_^−^; this difference in effect was significant (*P* = 0.003). Peripheral colonies directly on the surface of the bead (depth, 0 μm) were excluded from the statistical analysis as these colonies had an ample supply of O_2_.

### Size and spatial structure of aggregates in alginate beads.

**FIG 4:**
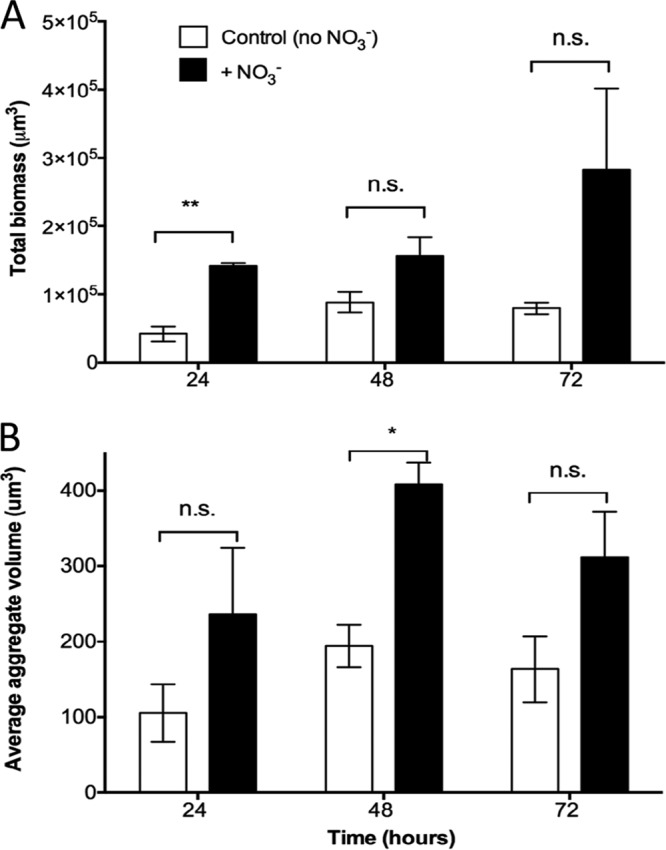
(A) Growth of alginate-encapsulated GFP-tagged P. aeruginosa as determined by quantification of total biomass in control beads (no NO_3_^−^) and NO_3_^−^-supplemented beads over time. Total biomass was quantified as the number of green fluorescent voxels. (B) Average P. aeruginosa aggregate volumes in alginate beads over time. Bars represent averages ± standard errors of the means from three replicates. In each group, >1,000 aggregates were analyzed. n.s., not significant; *, *P* < 0.05; **, *P* < 0.001.

**FIG 5:**
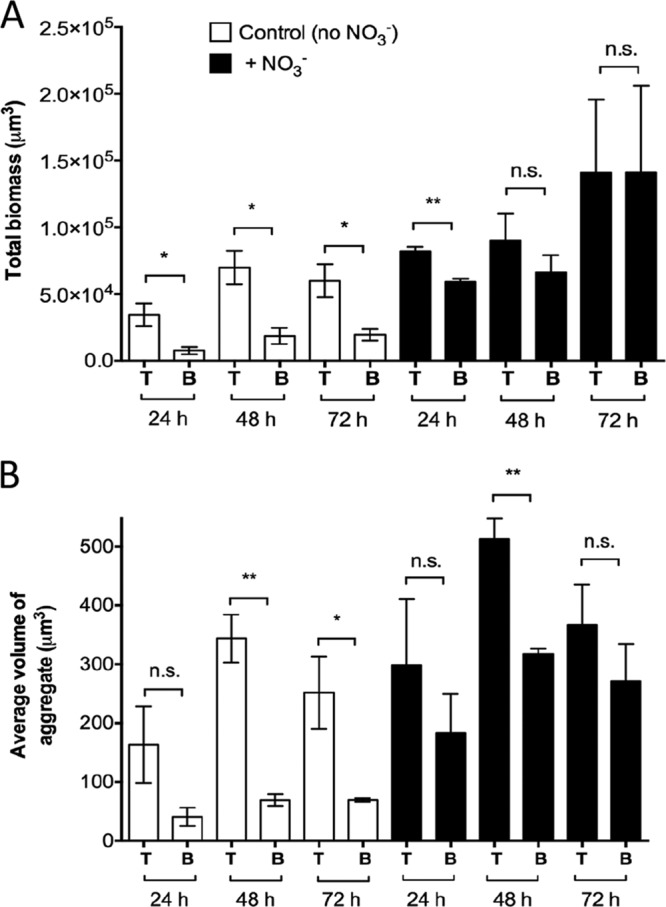
Spatial structure of alginate-encapsulated GFP-tagged P. aeruginosa PAO1. (A) Total biomass in the top (T) part, near the surface of the bead, and bottom (B) part of the image representing deeper parts of the beads. (B) Average aggregate volumes in the top (T) and bottom (B) parts of the images. The images were split in half at approximately 106 μm from the surface of the bead across the *x* axis. Bars represent averages ± standard errors of the means from three replicates. n.s., not significant, *, *P* < 0.05; **, *P* < 0.001.

When considering total aggregate volume in the beads, we generally found an average aggregate volume that was higher in beads supplemented with NO_3_^−^ (Fig. 4A), but this difference was only significant after 48 h (*P* = 0.0059). The same pattern applied to the total biomass (Fig. 4B), but here, the difference was only significant after 24 h (*P* = 0.0012). To assess the suitability of the alginate bead model for mimicking aggregate size as observed in chronic infections, we used area measurements (rather than volume) as data on CF lung tissue and chronic wound samples are only available in two dimensions (2D). The average (± standard deviation [SD]) cross-sectional areas of P. aeruginosa aggregates in the bead model after 24 h of growth were 77 μm^2^ ± 59 μm^2^ and 175 μm^2^ ± 100 μm^2^ in the absence and presence of NO_3_^−^, respectively. To determine the spatial structures of aggregates in the beads, we looked at the aggregates closest to the bead surface (T [top]) and compared them with aggregates deeper in the beads (B [bottom]). Our results revealed that significantly more biomass was situated in the bead periphery at each time point without nitrate (Fig. 5A) (*P* = 0.0394, 0.0213, and 0.0362 at 24, 48, and 72 h, respectively), but in the NO_3_^−^-supplemented beads, the differences disappeared after 24 h, resulting in an almost equal distribution of biomass after 72 h. Furthermore, we observed significantly larger aggregates in the bead periphery after 48 and 72 h of growth (*P* = 0.0028 and *P* = 0.0412, respectively) in beads without NO_3_^−^ (Fig. 5B). In the NO_3_^−^-supplemented beads, aggregate sizes were more evenly distributed throughout the beads over time, except at 48 h (*P* = 0.0058) (Fig. 5B).

### Respiration rates and O_2_ distribution.

**FIG 6:**
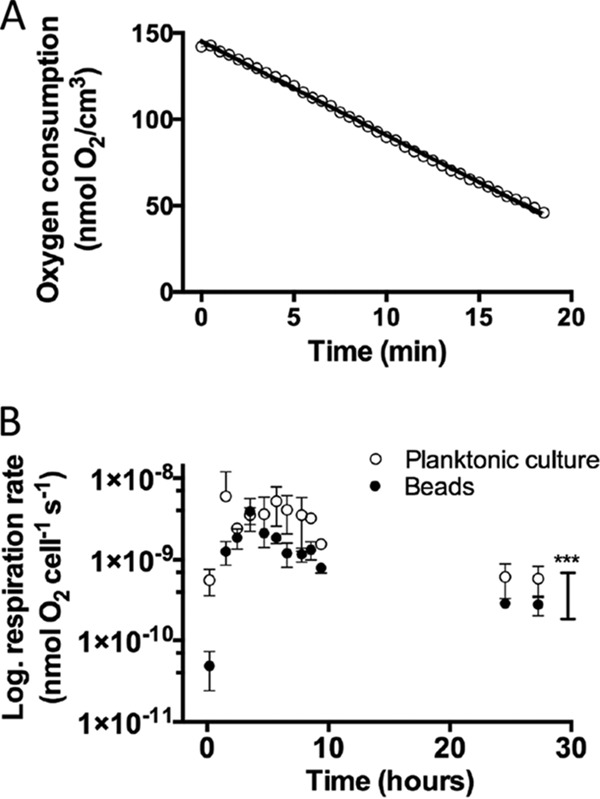
Respiration rate of P. aeruginosa PAO1. (A) Linear decrease in O_2_ concentration over time exemplified by the respiration rate of alginate-encapsulated P. aeruginosa after 4 h 50 min growth (*r*^*2*^ = 0.998; *P* < 0.0001). (B) Volumetric respiration rates, *R*, for alginate-encapsulated and planktonic P. aeruginosa calculated from the change in O_2_ concentrations at different time points during the experiment. Bars represent averages ± standard errors of the means from three or four replicates. ***, *P* < 0.0001.

O_2_ measurements showed a linear decrease in O_2_ concentration over time (Fig. 6A) (*r*^2^ = 0.99, *P* < 0.0001). Respiration rates of P. aeruginosa grown planktonically at 100 and 180 rpm were significantly higher (ρ = 0.46 ± 0.21 nmol O_2_ cell^−1^ s^−1^, *P* < 0.001 and ρ = 0.29 ± 0.21 nmol O_2_ cell^−1^ s^−1^, *P* = 0.012, respectively) than the volumetric respiration rate of alginate-encapsulated P. aeruginosa grown at 100 rpm (Fig. 6B). There was no significant difference between P. aeruginosa grown planktonically at 100 rpm and 180 rpm. Beads were not grown at 180 rpm due to mechanical rupture.

**FIG 7:**
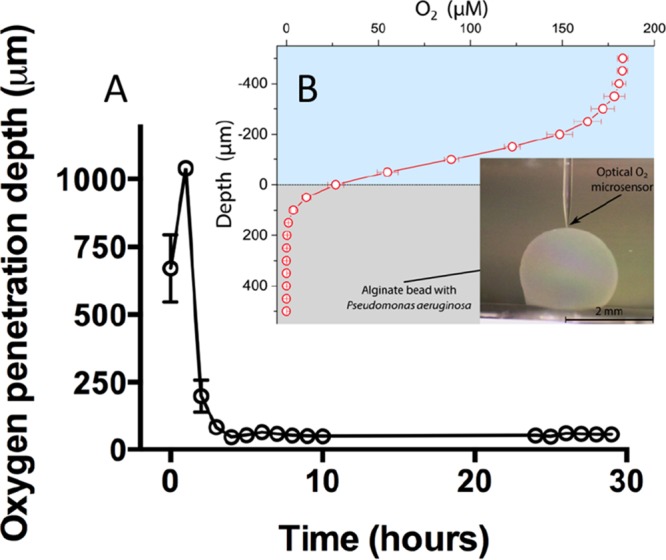
Oxygen penetration depth in alginate beads with P. aeruginosa. (A) Calculated O_2_ penetration depth (μm) during the time course of the experiment. Bars represent averages ± standard errors of the means from four replicates. Calculations were performed on the background of respiration rate measurements. (B) O_2_ microsensor profiles of P. aeruginosa grown in alginate beads for 24 h. The depicted profile is an average from six profiles obtained on three independent beads. Bars represent averages ± standard deviations. The inset shows the optical O_2_ microsensor touching the surface of the bead. All measurements were performed at 37°C.

We estimated an average O_2_ penetration depth of ∼50 μm in the alginate beads during 5 to 24 h of growth (Fig. 7A). To verify our calculated O_2_ penetration depth based on the respiration rate measurements, we conducted fiber-optic O_2_ microsensor profiling in similar beads, which confirmed that O_2_ was depleted within 50 to 100 μm from the surface of the bead (Fig. 7B).

### Expression profiles.

In support of the fact that alginate-encapsulated bacteria display a significantly lower volumetric respiration rate than their planktonic counterpart and physiological zonations are present in the beads due to steep O_2_ gradients, we performed transcriptional profiling. Profiles were obtained from planktonic P. aeruginosa and alginate-encapsulated P. aeruginosa grown in culture flasks shaken at 100 rpm for 24 h at 37°C with and without NO_3_^−^ supplementation. When comparing expressional profiles of alginate-encapsulated P. aeruginosa to a planktonic reference, 170 genes exhibited a >3-fold change in expression (see Table S2 in the supplemental material), with 17 upregulated and 153 downregulated genes. Some of the most notable upregulated genes were *ibpA* and the Anr-regulated genes *arcDABC*, *uspK*, and *uspN* (see Table S2 for roles and descriptions). When comparing alginate-encapsulated P. aeruginosa to an alginate-encapsulated reference with NO_3_^−^ supplementation, 141 genes were >3-fold differentially expressed, with 29 exhibiting upregulation and 112 showing downregulation. Besides the previously mentioned upregulated genes, we found *oprG* and the *ccON2*-encoded gene to be >3-fold induced (data not shown).

**FIG 8:**
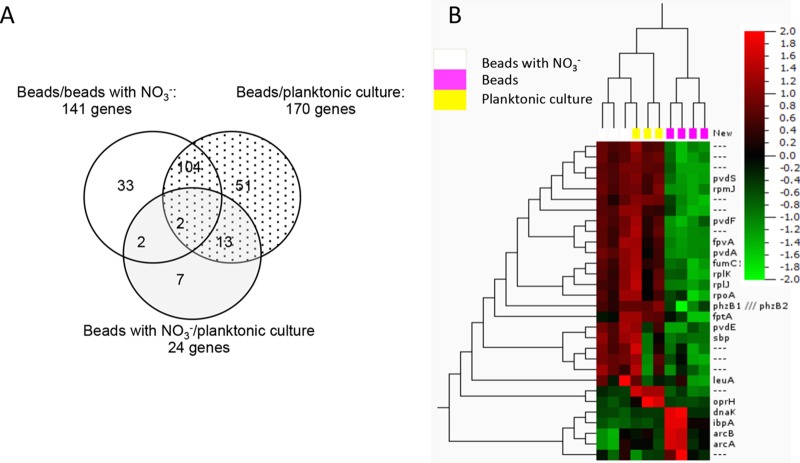
Differentially regulated genes, including those >3-fold up- and downregulated, in comparisons among the three *in vitro* conditions investigated with microarray. (A) Venn diagram comparing the genetic expressions of alginate-encapsulated P. aeruginosa (beads) with those of a planktonic reference culture of P. aeruginosa (dotted), where 170 genes showed >3-fold differential expression. When comparing alginate-encapsulated P. aeruginosa with and without NO_3_^−^ (white), 141 genes were >3-fold differentially expressed, and 104 of the genes were identical with those differentially regulated when comparing beads to planktonic culture. When comparing alginate-encapsulated P. aeruginosa with NO_3_^−^ with a planktonic reference culture (gray), just 24 genes showed differential expression of >3-fold. The differential expression of 2 genes (PA0456 and PA1869) was shared between all three subsets of comparisons (see Table S2, highlighted in gray). (B) Heat map of microarray data from alginate-encapsulated P. aeruginosa with and without NO_3_^−^ and a planktonic culture. The relative gene expressions are depicted according to the color scale shown in the top right corner.

Among the 141 genes, a total of 104 genes (underlined in Table S2) were identical to the 170 genes from the first comparison (Fig. 8A). Accordingly, when comparing the profiles of the two previously employed references (alginate-encapsulated P. aeruginosa supplemented with NO_3_^−^ versus planktonic culture) we found similar genetic expressions between the two (Fig. 8A and B), as only 24 genes were differentially expressed >3-fold. Two genes with >3-fold downregulation were shared among all three comparison subsets, namely, PA0456 (probable cold shock protein) and PA1869 (probable acyl carrier protein) (Fig. 8A) (highlighted in gray in Table S2).

None of the genes related to denitrification were induced >3-fold when comparing alginate-encapsulated P. aeruginosa supplemented with NO_3_^−^ to any of the other conditions, but we did find a moderate induction of *narK1* and *narI* (∼2-fold) when comparing the NO_3_^−^-supplemented beads to the reference without NO_3_^−^, and likewise, when comparing to the planktonic reference, we found an ∼2.5-fold induction of *narK1* and *narI* and an ∼2-fold induction of *norB*.

The downregulated genes were involved in translation, posttranslation, and degradation, predominantly genes encoding ribosomal proteins in the 30S and 50S subunits of the 70S ribosome (*rpm*, *rpl*, and *rps*) (framed in Table S2). Furthermore, we found a broad repression of genes associated with iron regulation. The following genes, all regulated by the ferric uptake regulator (Fur), were repressed in alginate-encapsulated P. aeruginosa in comparison to the planktonic reference: sigma factor PvdS PA2426 (*PvdS*), ferri-siderophore receptor genes PO2398 (*fpvA*) and PA4221 (*fptA*), siderophore (pyochelin) biosynthesis genes PA4226 (*pchE*) and PA4228 to PA4231 (*pchDCBA*), and siderophore (pyoverdine) system-related genes PA2386 (*pvdA*), PA2394 (*pvdN*), PA2396 to PA2399 (*pvdFEAD*), and PA2401 (*pvdJ*) (see Table S2).

### Antibiotic tolerance.

**FIG 9:**
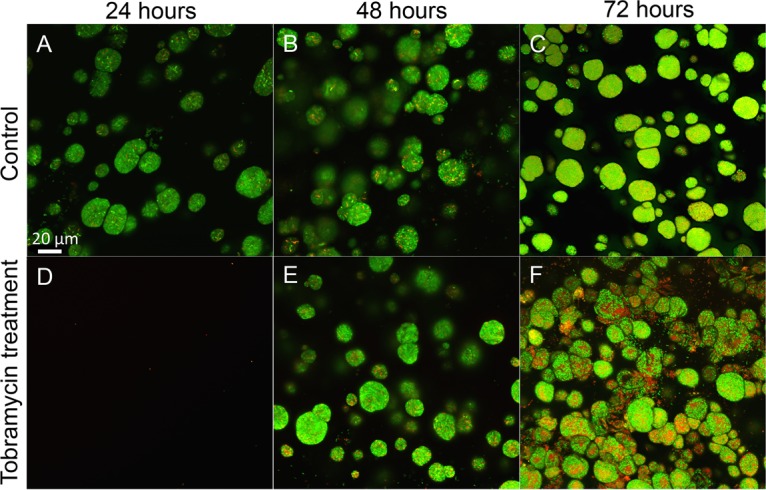
Antibiotic tolerance of alginate-encapsulated P. aeruginosa PAO1. (A to C) CLSM images of nontreated control beads with P. aeruginosa grown for 24, 48, and 72 h. (D to F) CLSM images of alginate-encapsulated P. aeruginosa treated with 100 μg/ml tobramycin for 24 h. (D) P. aeruginosa exposed to tobramycin just after encapsulation (when still in single-cell planktonic state). P. aeruginosa was allowed to form aggregates for 24 h (E) or 48 h (F) before the exposure to tobramycin. Tobramycin exposure lasted for 24 h. Notice how the majority of the bacteria are green (alive) in spite of 24-h antibiotic treatment. Viability staining was with Syto9 and PI. Green bacteria are alive and red/yellow bacteria are dead. Bar, 20 μm.

Observation by confocal laser scanning microscopy (CLSM) and viability staining with Syto9 and propidium iodide (PI) revealed that alginate-encapsulated P. aeruginosa cells were susceptible to tobramycin at 100× the MIC (100 μg ml^−1^) immediately after encapsulation, when the bacteria were still in a planktonic state (Fig. 9D). However, when allowed first to grow for 24 or 48 h, P. aeruginosa prevailed for 24 h in the presence of tobramycin at 100× the MIC (100 μg ml^−1^) (Fig. 9E and F).

## DISCUSSION

The goal of this study was to develop an *in vitro* model system that recapitulates the physical aspects of nonattached aggregate growth observed in chronic infections and provides a versatile platform for studying bacterial aggregates. The model uses alginate-encapsulated (24, 25) P. aeruginosa, mimicking growth conditions under diffusion limitation through the secondary matrix as seen in chronic infections (26). By incorporating the alternative electron acceptor NO_3_^−^ into the beads, we mimicked and studied the anaerobic growth patterns in P. aeruginosa. To establish the relevance of the model, we compared the observed aggregate sizes to previous measurements of P. aeruginosa aggregates in CF lungs and chronic wounds. Kragh et al. (17) analyzed 59 biofilms from 20 *ex vivo* lung tissue samples from three CF patients and found the areas of biofilm aggregates ranged from 4 to 3,227 μm^2^, and we found the areas of aggregates to range from 23 to 342 μm^2^ (24 to 438 μm^2^ when supplemented with NO_3_^−^), which is within the same range. In a review by Bjarnsholt et al. (1), the diameters of aggregates in a range of chronic infections were measured and were found to be within 5 and 50 μm for the smallest and largest biofilms, respectively (∼20 to 2,000 μm^2^, respectively, when assuming the aggregates were spherical). Thus, alginate-encapsulated P. aeruginosa had an aggregate size (Fig. 4A) within the same range found in CF lungs (17) and other chronic infections (1). In addition, the aggregates were separated by a secondary matrix similar to what is observed in the lungs of CF patients (Fig. 1B), and there was no attachment to a surface.

We found steep O_2_ concentration gradients in the alginate beads (Fig. 7A and B), which is also a recognized feature in chronic infections (18, 27–29). The aggregates formed primarily in the outermost ∼100 μm of the beads (Fig. 2A), corresponding to the oxygenated zone where larger aggregates and, hence, a larger proportion of the biomass, are situated in comparison to deeper sites (Fig. 5A and B). This, together with the diffusion-limited supply of O_2_, led to a strong depletion of this preferred electron acceptor in the periphery of the alginate beads (30). A comparable O_2_ distribution was reported by Walters et al. (31), who found an O_2_ penetration depth of 50 to 90 μm into dense bacterial colonies, while Pabst et al. (32) found a similar heterogeneous bacterial distribution when studying gel-entrapped Staphylococcus aureus. In the CF lung, P. aeruginosa can grow in hypoxic/anaerobic mucus (18), which may be supported by the alternative electron acceptor NO_3_^−^ (33). We alleviated electron acceptor limitation by adding 100 mM NO_3_^−^ to the system, which resulted in a more homogenous growth and extended distribution of bacterial aggregates within the beads (Fig. 2B). After 72 h of growth with the NO_3_^−^ supplement (Fig. 5A), the observed difference between the total biomasses in the top and bottom halves disappeared, indicating that peripheral growth was indeed a result of O_2_ limitation in the absence of NO_3_^−^, and supplying NO_3_^−^ as an alternative electron acceptor reduced P. aeruginosa's need to be positioned in oxygenated zones. The use of 100 mM NO_3_^−^ in the experiments is based on previous studies, where 100 mM NO_3_^−^ was shown to yield high cell densities (34, 35). The concentrations of NO_3_^−^ reported from CF sputum rarely exceed 1 mM (20, 33); however, the use of 100 mM NO_3_^−^ may be argued against as measurements of NO_3_^−^ concentrations in CF lungs are based on bulk measurements or homogenates of sputum, meaning that niches containing high concentrations of NO_3_^−^ may exist.

The estimated apparent growth rates of P. aeruginosa in alginate beads decreased with time and with increasing depth into the beads (Fig. 3), but such growth limitation due to O_2_ depletion was alleviated by NO_3_^−^ supplementation. These observations in the alginate bead model support previous speculations that the availability of NO_3_^−^ contributes to the apparent lack of internal gradients of growth in P. aeruginosa biofilm aggregates in the endobronchial mucus of CF patients with chronic lung infections (18), where O_2_ is restricted by the intensive oxygen consumption by activated PMNs (14, 33). Furthermore, our finding that NO_3_^−^ supplementation sustained the growth of P. aeruginosa microcolonies under O_2_ depletion may explain *in vivo* findings of P. aeruginosa residing deeper within wound beds of patients suffering from chronic wounds (11). In this respect, the lower concentrations of NO_3_^−^ and NO_2_^−^ observed in infected wounds compared with those in noninfected wounds (36) suggest consumption via bacterial denitrification. The respiration rate of alginate-encapsulated P. aeruginosa was significantly lower than in their planktonic growth mode; respiration rates were of similar magnitude as those observed in other studies of planktonic bacteria (37, 38). Alginate-encapsulated bacteria were less metabolically active due to steep O_2_ gradients within the alginate beads, a fact that was supported by our transcriptional analysis. The upregulation of the Anr-controlled genes PA3309 (*uspK*), PA4352 (*uspN*), PA5170 to PA5173 (*arcDABC*), PA4067 (*oprG*), and PA1557 (*ccON2*-encoded gene) in alginate beads corresponds to results from previous studies showing that these genes were highly expressed in P. aeruginosa biofilms (39, 40) and were predominantly associated with metabolism, O_2_ limitation, anaerobic survival, and stationary-phase growth (34, 41). Anr is a key regulator that induces the expression of genes during hypoxia and can be regarded as a marker for hypoxic or anaerobic growth (41). However, the Anr regulon itself was not upregulated, in line with the finding of Alvarez-Orgeta et al. (34) that a change in the transcriptional level of the Anr regulon is not in itself an essential component in the response to low O_2_. The 10-fold induced gene PA3126 (*ibpA*) (42–45) encodes a protein with high similarity to the Escherichia coli heat shock protein IbpA, which is usually not induced during anaerobiosis in E. coli (41, 46), but is recognized as a responder to low oxygen in other bacterial species (47). In the absence of O_2_ and in the presence of NO_3_^−^ or NO_2_^−^, P. aeruginosa can grow by denitrification (48). Thus, we expected an induction of the nitrate reductase genes (*narGHJI*) (20) when comparing genetic expression profiles from NO_3_^−^-supplemented beads to profiles from beads without NO_3_^−^. Surprisingly, nitrate reductase genes were not induced >3-fold in the NO_3_^−^-supplemented beads, but according to Alvarez-Ortega et al. (34), the elevation of nitrate reductase genes is not indicative of anaerobic denitrification. In fact, P. aeruginosa may upregulate denitrification genes as a response to low oxygen irrespective of NO_3_^−^ availability. Furthermore, P. aeruginosa can sustain moderate anaerobic growth by arginine (49) and pyruvate fermentations, which do not support growth but facilitate long-term survival (50). We found that the alcohol dehydrogenase gene *adhA* was induced, which is indicative of fermentation (41), another important adaptation to a microanaerobic or anaerobic environment. The overall downregulation of genes involved in translation, posttranslational modification, and degradation is in concordance with the findings of Trunk et al. (41). Metabolically active and fast-growing cells synthesize ribosomes, and so a higher expression of ribosomes in a planktonic culture is expected. Williamson et al. (45) found the ribosomal proteins to be expressed >2-fold at the top of an *in vitro* biofilm compared with at the bottom, again supporting the idea that the alginate-encapsulated bacteria become O_2_ limited. The downregulation of stationary-phase sigma factor *rpoS* (45, 51) and quorum-sensing (QS) regulators *lasR* (∼2-fold) and *rhlR* (45) indicates low metabolic activity, which is further supported by the downregulation of PA4853 (*fis*) (43), a gene associated with early exponential growth. One of the most notable findings was the broad repression of genes associated with iron regulation, which is concordant with findings by Chang et al. (52) and James et al. (29). The general repression of iron regulation genes may be due to the iron-binding properties of alginate, thus concentrating iron from the growth medium in the alginate beads over time (53). PA4468 (*sodM*) and PA4470 (*fumC*) were also downregulated, which is concordant with the general repression of genes related to iron limitation, as *sodM* (34) and *fumC* (54) are only activated in cases of iron deprivation. All in all, this is supportive of our findings that alginate-encapsulated P. aeruginosa experiences a lower respiration rate than its planktonic counterpart due to O_2_ limitation, resulting in the expression of genes associated with hypoxia stress and low metabolic activity.

The alginate bead model displayed a characteristic biofilm-associated tolerance toward tobramycin. Newly embedded active P. aeruginosa in the “planktonic state” was susceptible to tobramycin and eradicated upon treatment (Fig. 9D). This confirmed that tobramycin is capable of penetrating the alginate beads (55) and that the effect of tobramycin is dependent on the physiological growth stage of P. aeruginosa rather than on transport limitation. While the underlying reasons remain elusive, one hypothesis could be that the hypoxic conditions and low respiration rate of alginate-encapsulated bacteria antagonize the effect of tobramycin (31), and thus, the increased tolerance toward antibiotics is in part due to the differences in physiological and metabolic growth stages (56). We found that the genetic expression profiles of planktonic bacteria and those of alginate-encapsulated bacteria supplemented with NO_3_^−^ were highly similar, as depicted graphically in a Venn diagram and heat map (Fig. 8A and B). This suggests that genetic expression in the latter case was not affected by the aggregated state but rather by alleviating the electron acceptor limitation with NO_3_^−^. Furthermore, antibiotic tolerance is a reversible state, as the antibiotic susceptibility can be restored if bacteria are released from biofilms (31) or are reoxygenated (57).

Collectively, the results provide insight into the physiochemical environment of nonattached aggregates and an alternative to surface attachment models. The model recapitulates the physical aspects of microbial biofilms in terms of antibiotic tolerance, heterogeneous growth, which was alleviated by adding NO_3_^−^, and hypoxia, as confirmed by microsensor measurements and transcriptional analysis. With the alginate bead model, it is thus possible to mimic *in vivo* chronic infections, thereby helping to bridge the gap between *in vitro* and *in vivo* biofilms.

## MATERIALS AND METHODS

### Bacterial strains and media.

The P. aeruginosa strain PAO1 was obtained from the Pseudomonas Genetic Stock Center at East Carolina University and was used in all experiments. A stable green fluorescent protein (GFP) constitutively expressed by plasmid pMRP9 (58) was used to tag the bacteria. Overnight (ON) cultures were propagated from −80°C frozen culture stocks and grown overnight in lysogeny broth (LB) for ∼18 h at 37°C under continuous shaking at 180 rpm. The LB ON culture was subsequently used for inoculation in low-nutrition R2A broth (Lab M Ltd, UK) supplemented with 0.05 M Tris-HCl buffer (pH 7.6) and 0.5% glucose (abbreviated R2A), and was left to acclimatize ON until further use. The medium-to-volume ratio was 1:2.5.

### Bead preparation.

The encapsulation of P. aeruginosa in alginate beads was performed using a modification of the methods by Pedersen et al. (59) and Behrendt et al. (24). Autoclaved seaweed alginate (2% [wt/vol]) (Protanal LF 10/60 FT; FMC Biopolymer, Norway) was dissolved in milli-Q water with or without the addition of 100 mM potassium nitrate (KNO_3_) (P8394; Sigma-Aldrich, USA) (34, 35). An ON culture of P. aeruginosa in R2A was adjusted to a final optical density at 450 nm (OD_450_) of 0.1 in alginate. Droplets of the alginate with bacteria were dispensed via a 21-gauge needle placed 3 cm above the surface of a stirred 0.25 M CaCl_2_ solution, wherein the beads were hardened for 1 h. This procedure was previously reported to yield spherical and stable beads (60). We produced nearly uniform spherical beads of 2.4 ± 0.1 mm (mean ± SD) with this procedure. Hardened beads were rinsed in 0.9% NaCl before being transferred to prewarmed R2A media. In all experiments, beads were incubated in R2A at 100 rpm at 37°C, unless otherwise mentioned.

### Viable cell counts.

To release the bacteria, beads were dissolved using a solution of Na_2_CO_3_ and citric acid (61), which were mixed in equal amounts before use to yield final concentrations of 0.05 and 0.02 M, respectively. Solubilized beads or planktonic cultures were degassed and sonicated for 5 min, serially diluted, and plated on LB plates for the enumeration of cells by colony formation.

### Microscopy and image analysis.

For image analysis of the spatial organization and growth of bacterial aggregates, alginate-encapsulated P. aeruginosa was grown with and without NO_3_^−^ supplementation and was sampled in triplicates after 24, 48, and 72 h of growth. Beads were cut in half, and images were acquired with CLSM (Zeiss.Z2; LSM 710, Germany) of the cut surface with an emphasis on visualizing the edge of the bead and as much of the bead interior as possible. Images were recorded as z-stacks in 1-μm increments with a 40×/1.3 numerical aperture (NA) oil immersion objective. Image analysis was performed using Imaris v8.3.1 (Bitplane, Switzerland). To calculate 2-dimensional cross-sectional areas of aggregates in the beads after 24 h of growth with and without NO_3_^−^, we excluded aggregates <10 μm^2^ and aggregates touching the edge of the image to avoid planktonic bacteria and incomplete aggregates. To elucidate whether the ability to grow anaerobically impacted the spatial structure and distribution of the bacterial aggregates in beads with and without NO_3_^−^, the images were split in half ∼106 μm from the surface of the bead across the *x* axis to separate the top (T) half from the bottom (B) half of the image, and T and B were compared statistically. Total biomass (all voxels detected), average aggregate volume (object volume), and z positions were calculated with the ImarisVantage module. The total biomass was calculated by first subtracting background fluorescence from all 3-dimensional image stacks. The background fluorescence in the green channel was calculated by creating histograms of three different areas of the edge of the image, and the highest voxel value was determined. The three values were then averaged and a value of 92 voxels was determined as the background fluorescence. Isosurfaces were created of the remaining voxels and the sum of all individual objects was used to calculate the total biomass. The average aggregate volume was also calculated.

Imaging of the alginate bead sections for quantitative PNA-FISH was performed with the same settings used for acquiring all the pictures. Fluorescence images were recorded as 1-μm z-stacks at a resolution of 4,096 by 4,096 pixels, with an averaging of 2 at 16-bit color depth, using a 63×/1.4 NA oil immersion objective and 594 nm laser excitation. Microcolony fluorescence was quantified using ImageJ (National Institutes of Health, USA) using a previously described procedure (17). Colony distances from the periphery of alginate beads were determined with the measuring tool in the microscope image analysis software (Zen2010, version 6.0; Zeiss, Germany).

### Quantitative PNA-FISH.

Alginate-encapsulated P. aeruginosa was grown with and without NO_3_^−^-supplemented alginate and medium, was sampled chronologically after 12, 24, 48, and 72 h, and was stored at 4°C in 4% formalin (Hounisen, Denmark) with 0.25 M CaCl_2_ for stabilization. The beads were embedded in paraffin, cut in 4-μm sections with a standard microtome, fixed on glass slides, and kept in the dark at 4°C until further treatment. The sections were deparaffinized and stained with a Texas Red-conjugated 16S rRNA probe (AdvanDx, USA) specific for P. aeruginosa as previously described (17). To stabilize the samples prior to staining, one drop of GN fixation solution (AdvanDx, USA) was applied to each sample and left for incubation at 65°C for 20 min. The slides with alginate bead sections were washed in wash solution (AdvanDx, USA) at 55°C for 30 min, air dried briefly, and then one drop of ProLong Gold antifade reagent (Life Technologies, USA) and a coverslip were applied.

### Respiration rate measurements.

Molecular oxygen concentrations were measured with O_2_-sensitive optode sensor spots (37, 62) mounted with silicon glue on the inside of air-tight cuvettes (35 mm by 12 mm culture tubes; schuett-Biotec, Germany) and monitored through the transparent cuvette wall with a 2-mm fiber-optic cable connected to a fiber-optic O_2_ meter (Fibox 3; PreSens GmbH, Germany). The optodes were calibrated (in units of μmol O_2_ liter^−1^) by a two-point calibration procedure before each experiment using measurements in air-saturated and O_2_-free R2A at the experimental temperature (37°C) and pH (7.6).

For respiration rate measurements on alginate-encapsulated bacteria, beads were drawn with a transfer pipette from the culture flask, rinsed 3 times with prewarmed 0.9% NaCl, and then transferred to a cuvette filled with prewarmed (37°C) sterile R2A and a glass coated magnet. The cuvette was closed air tight, mounted on a magnetic stirrer, and fitted with the fiber-optic readout cable. Each measurement followed the O_2_ depletion in the cuvette over time, and the total respiration rate of the beads was calculated from linear parts of the declining O_2_ concentration versus time curve (in units of μmol O_2_ liter^−1^ h^−1^). Respiration rates of planktonic bacteria were measured in a similar way using planktonic bacteria grown at two different flow speeds, namely, 100 rpm (similar to the beads) and 180 rpm (standard for planktonic cultures). If active cells do not exhibit a homogenous distribution in the beads, the respiration rate (*R*) will be underestimated. We compensated for the heterogeneous distribution of bacterial cells due to the clustering of bacterial aggregates in the periphery of the beads by using the calculated values of *r* (radial distance encompassing the bacterial growth band) at the different time intervals to recalculate *R* as the volumetric respiration rate (see section S1, equation 10, in the supplemental material). The O_2_ penetration depth in the alginate beads, *r*, was calculated from the measured cell density and concentration of O_2_ at the surface of the beads, *C_0_* (see section S1, equation 14).

Respiration measurements were performed hourly during the first 8 h and the experiments lasted ∼30 h. The respiration rate experiments were conducted on 4 biological replicates, and bacterial cell counts within each experiment were performed in duplicates. Total respiration rates were combined with quantifications of bacterial numbers and growth zonations to estimate cell-specific, *R_cell_*, and bead volume-specific, *R*, respiration rates by using simple diffusion-reaction relations for a spherical geometry as outlined in the supplemental material.

### Microsensor measurements.

A single bead was submerged in a petri dish filled with R2A after 24 h of growth in a culture flask. The petri dish was placed on a heated plate (set to 37°C) and gently aerated by a fine air stream directed toward the surface via a Pasteur pipette connected to an air pump. A fiber-optic O_2_ microsensor (OXR50-HS; tip diameter, 50 μm) was mounted on a motorized micromanipulator (MU1) and connected to an O_2_ meter (FireStingO2); all components were obtained from Pyro-Science GmbH, Germany. Calibration of the microsensor was performed as specified by the manufacturer via measurements in air-saturated and O_2_-free medium. The position where the sensor touched the bead (depth, 0) was determined visually with the help of a USB microscope (model AM7515MZTL; Dino-Lite). Microsensor positioning and data acquisition were performed with dedicated profiling software (Profix; Pyro Science GmbH, Germany). Data were analyzed in Origin Pro 9.0.

### Microarray analysis.

RNA was isolated from stationary-phase planktonic and alginate-encapsulate*d* P. aeruginosa after 24 h of growth with and without NO_3_^−^. For alginate bead cultures, the beads were harvested and rinsed three times in sterile, prewarmed 0.9% NaCl to remove planktonic bacteria before mixing with two volumes of RNAlater (Ambion, USA). The samples were stored ON at 4°C before freezing at −80°C until further use. To dissolve the alginate beads before RNA isolation, the frozen beads were thawed at 4°C and ultrasound (Sonoca Söring GmbH, Germany) was administered at the lowest intensity until the alginate beads appeared completely dissolved. Cells were harvested by centrifugation at 7,000 × *g* for 15 min at 4°C. The supernatant was removed, and the cell pellet lysed with 100 μl 1 mg ml^−1^ lysozyme (Sigma-Aldrich, USA) at room temperature for 13 min. RNA isolation was performed with an RNeasy mini purification kit (Qiagen, Netherlands), and contaminating chromosomal DNA was removed by RQ1 RNase-free DNase treatment (Promega, USA). RNA quality and quantity were detected with a NanoDrop spectrophotometer (Fischer Thermo Scientific, USA). cDNA synthesis and hybridization were performed by the Microarray Center at the Copenhagen University Hospital (Denmark), and the arrays were scanned in the Affymetrix GeneArray 3000 7G scanner. Cell intensity files (CEL files) were generated in the GeneChip Command Console software (AGCC) (Affymetrix, USA). Gene expressions were analyzed using the software Arraystar (version 3.0; DNAstar, USA).

### Antibiotic tolerance.

Antibiotic tolerance of alginate-encapsulated P. aeruginosa was investigated by challenging the beads with 100× the MIC of tobramycin for P. aeruginosa (100 μg ml^−1^ tobramycin) (6, 10) in the growth medium at different growth stages, at 0, 24, and 48 h after alginate encapsulation. Beads were incubated with antibiotics for 24 h and subjected to live/dead staining with Syto9 (Life Technologies, Waltham, MA, USA) and propidium iodine (PI) (Sigma-Aldrich, USA) to evaluate the antibiotic effect visually by CLSM (Zeiss.Z2 LSM 710).

### Statistical analysis.

Data were analyzed for statistical significance with SPSS 22 software (IBM, USA) and illustrated in GraphPad Prism 6 software (GraphPad software, USA) and Origin Pro 9.0 (Origin Lab, USA). Respiration rate data were analyzed by linear mixed models. Quantitative PNA-FISH data were compared by a linear regression analysis, a Mann-Whitney U test, and an independent *t* test. Aggregate volumes and biomass were analyzed by multiple *t* tests. A *P* value of <0.05 was considered significant.

## Supplementary Material

Supplemental material
