# Supplementary material for: Pseudomonas aeruginosa Aggregate Formation in an Alginate Bead Model System Exhibits In Vivo-Like Characteristics
Source: Appl Environ Microbiol. 2017 Apr 17;83(9):e00113-17. doi: 10.1128/AEM.00113-17 (PMC5394317; doi:10.1128/AEM.00113-17)
Supplement: Supplemental material [file supp_83_9_e00113-17__index.html]

Pseudomonas aeruginosa Aggregate Formation in an Alginate Bead Model System Exhibits In Vivo-Like Characteristics — Supplemental material 

# Pseudomonas aeruginosa Aggregate Formation in an Alginate Bead Model System Exhibits *In Vivo*-Like Characteristics

## Supplemental material

- Supplemental file 1 -

  Mathematical models for respiration rate measurements; microarray data (Table S2).

  PDF, 2.5M
