# Supplementary material for: Pseudomonas aeruginosa Aggregate Formation in an Alginate Bead Model System Exhibits In Vivo-Like Characteristics
Source: Appl Environ Microbiol. 2017 Apr 17;83(9):e00113-17. doi: 10.1128/AEM.00113-17 (PMC5394317; doi:10.1128/AEM.00113-17)
Supplement: Supplemental material [file AEM.00113-17_zam999117808s1.pdf]

Supplemental material S1

Mathematical models for respiration rate measurements

We measured a change in O_2 concentration, *C* (µmol l^{-1} = nmol cm^{-3}) over time, *t* (s), where *W* (cm^3) is the volume of the surrounding medium in the incubation vial:

$$\frac{dC}{dt} \times W \ (nmol\ O_2\ s^{-1}) \quad (1)$$

In a filled cuvette with planktonic bacteria the volume is described as

$$V \ = V_{magnet} + W \quad (2)$$

and in a filled cuvette containing *n* beads the volume from which the change in O_2 concentration is measured is

$$V \ = V_{beads} + V_{magnet} + W \quad (3)$$

A cuvette with total volume *V* (cm^3) contains *n* individual beads each with a radius r_0 (cm). The total bead volume is thus described by

$$V_b \ = n \times \frac{4}{3}\pi r_0^3 (cm^3) \quad (4)$$

and the total surface area is described by

$$A_b \ = n \times 4\pi r_0^2 (cm^2) \quad (5)$$

Thus, the O_2 consumption pr. bead is given by

$$\frac{\left(\left(\frac{dc}{dt}\right) \times W\right)}{n} \ (nmol\ O_2\ bead^{-1}\ s^{-1}) \quad (6)$$

and the cell-specific O_2 respiration rate as

$$\frac{\left(\frac{dC}{dt} \times W\right)\Big/_{n}}{N} \ (nmol\ O_2\ cell\ s^{-1}) \quad (7)$$

where *N* is the number of bacterial cells in each alginate bead.

The O_2 consumption pr. surface area pr. bead is given by

$$R(A) = \frac{\left(\frac{dC}{dt} \times W\right) \Big/ n}{4\pi r_0^2} \; (nmol\ O_2\ cm^{-2}\ s^{-1})$$

(8)

and the O_2 consumption pr. volume bead is given by:

$$R = \frac{\left(\frac{dC}{dt} \times W\right) \Big/ n}{\frac{4}{3}\pi r_0^3} \; (nmol\ O_2\ cm^{-3}\ s^{-1})$$

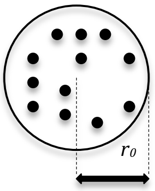

(9)

when assuming a homogenous distribution of active bacterial cells throughout the entire bead. If active bacterial cells do not exhibit a homogenous distribution in the bead, *R* will be underestimated. If active cells are clustering in the periphery of the beads, *R(V)* can instead be calculated as:

$$R = \frac{\left(\frac{dC}{dt} \times W\right)}{n\frac{4}{3}\pi(r_0^3 - r_n^3)}$$

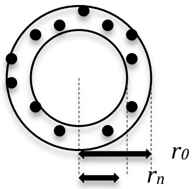

(10)

where r_0 *and* r_n denote the inner- and outer-most radial distance encompassing the bacterial growth band, i. e., the spherical shell in the bead containing active bacteria.

As a consequence of the respiration of the alginate encapsulated bacteria the O_2 concentration in the alginate bead decreases towards the centre. The O_2 concentration within a spherical cell aggregate at steady state can be described as (11):

$$C_r = -\frac{R}{6D_{agg.}}(r_0^2 - r^2) + C_0$$

(11)

where C_r is the oxygen concentration at the radial distance *r*, D_{agg} is the molecular diffusion coefficient in alginate at 37°C (here assumed to be similar to *D* in water (1) at 37°C; $D = 1.5\times10^{-5}$ $cm^2\ s^{-1}$), *R* is the bead volume-specific respiration rate and C_0 is the O_2 concentration at the surface of the bead. Here we assume that C_0 is the same as for the surrounding medium, due to the absence of an effective diffusive boundary layer, δ_{eff}, under the very turbulent environment in which the beads were kept during respiration measurements. In a

stagnant culture, the hypothetical δ_{eff} = r_0 and in a turbulent environment δ_{eff} decreases inversely proportionally with *Sh* (the Sherwood number) approaches 0 at high turbulence (1).

Assuming that the centre of the alginate bead is anoxic, the equation can be rearranged to the following allowing for the calculation of the O_2 penetration depth based on volumetric respiration rate and concentration of oxygen at the surface of the beads, C_0.

$$C_r = -\frac{R}{6D_{agg.}}(r_0^2 - r^2) + C_0 = 0$$

(12)

and

$$C_0 = \frac{Rr_0^2}{6D_{agg.}}$$

and

(13)

$$r = \sqrt{\frac{C_0 6D_{agg.}}{R}}$$

(14)

In a sphere, were an anoxic core is present because all the O_2 is consumed in the periphery of the sphere, the respiration rate needed to create anoxia at the radial distance *r* within a sphere is described by (1):

$$R = 3C_\infty \left(\frac{r_0^2 - r_n^2}{2D_{agg.}} + \frac{\delta_{eff}\ r_0}{D_w} - \frac{\delta_{eff}\ r_n^3/r_0^2}{D_w}\right)^{-1}$$

(15)

Again, by assuming that the effective boundary layer is non-existent under the experimental conditions, the equation can be re-arranged to the following, by replacing δ_{eff} with 0:

$$R = 3C_\infty \left(\frac{r_0^2 - r_n^2}{2_{agg.}}\right)^{-1}$$

(16)

By assuming that the theoretical maximum diffusive boundary layer, $\delta_{eff} = r_0$ the equation can be re-written as:

$$R = 3C_\infty \left(\frac{r_0^2 - r_n^2}{2D_{agg.}} + \frac{r_0^2}{D_w} - \frac{r_0 r_n^3/r_0^2}{D_w}\right)^{-1}$$

(17)

Equations (16) and (17) enable a theoretical estimate of R under the assumption of no δ_{eff} and a maximum δ_{eff}, respectively, and from the linear relationship between the two a hypothetical DBL can be calculated.

Supplemental material S2

Locus ID[a]	Gene name	Linear fold change[b]		Roles[c]	Description
		Beads/Planktonic	Beads/Beads-NO_3		
**Upregulated genes in alginate beads**					
PA1517[e]		3.02	3.88		Conserved hypothetical protein
PA1746		3.98	3.59	O_2 (1, 2), SP (2)	Hypothetical protein
PA2247	*bkdA1*	3.45	3.04	H_2O_2 (3)	2-oxoisovalerate dehydrogenase (alpha subunit)
PA2501		4.38	7.43	O_2 (1)	Hypothetical protein
PA2931	*cifR*	3.06	3.89	VF[d]	CifR
PA3006	*psrA*	5.82	6.48	O_2 (1)	Transcriptional regulator PsrA
PA3126	*ibpA*	10.61	15.70	O_2 (1), heat-shock[d]	Heat-shock protein ibpA
PA3309	*uspk*	5.37	4.42	O_2 (1, 2)	UspK pyruvate fermentation and long-term anaerobic survival
PA3531	*bfrB*	3.30	3.54		Bacterioferritin
PA3601		7.35	6.20		Conserved hypothetical protein
PA4352	*uspN*	3.70	3.77	O_2 (1, 2), SP (2)	UspN pyruvate fermentation and long-term anaerobic survival
PA4542	*clpB*	3.09	3.45	Heat-shock[d]	ClpB protein
PA4577		3.72	4.27	O_2 (1), SP (2)	Hypothetical protein
PA4761	*dnaK*	3.31	3.66	Heat-shock[d]	DnaK protein
PA5170	*arcD*	3.31	3.73	O_2 (1), SP (2)	Arginine/ornithine antiporter
PA5232		3.12	3.56	O_2 (1)	Conserved hypothetical protein
PA5427	*adhA*	3.66	4.13	O_2 (1, 2), SP (2)	Alcohol dehydrogenase
**Downregulated genes in alginate beads**					
PA0122[f]	*rahU*	-6.37	-1.31	HSL QS (4), SP (2)	
PA0139	*ahpC*	-3.75	-2.14	H_2O_2 (3)	Alkyl hydroperoxide reductase subunit C
PA0179		-5.61	-1.06	SP (2)	Probable two-component resonse-regulator
PA0258		-4.14	-2.77		Hypothetical protein
PA0264		-4.46	-4.24		Hypothetical protein
PA0283	*sbp*	-3.83	-10.85		Sulfate-binding protein precursor
PA0284		-17.93	-86.03		Hypothetical protein
PA0456[g]		-10.01	-3.08		Probable cold-shock protein
PA0579	*rpsU*	-3.75	-2.97		30S ribosomal protein S21
PA0668	*tyrZ*	-3.04	-1.93		Tyrosyl-tRNA synthetase 2
PA0769		-3.28	-2.19		Hypothetical protein

PA0805		-4.27	-3.48		Hypothetical protein
PA0971	*tolA*	-4.71	-4.25		TolA protein
PA1178	*oprH*	-14.13	-1.06	SP (2), Mg (5)	PhoP/Q and low Mg2+ inducible outer membrane protein H1 precursor
PA1245		-7.90	-7.97	SP (2)	AprX
PA1247	*aprE*	-4.96	-6.30		Alkaline protease secretion protein AprE
PA1248	*aprF*	-3.11	-4.21		Alkaline protease secretion outer membrane protein AprF precursor
PA1372		-4.92	-3.56		Hypothetical protein
PA1431	*rsaL*	-4.38	-3.30	SP (2), O_2 (2), HSL QS (4)	Regulatory protein RsaL
PA1533		-3.81	-2.94		Conserved hypothetical protein
PA1588	*sucC*	-4.28	-4.03		Succinyl-CoA synthetase beta chain
PA1774	*crfX*	-3.76	-2.53		CrfX protein
PA1776	*sigX*	-3.28	-1.95		ECF sigma factor SigX
PA1784		-3.06	-1.46	SP (2), HSL QS (4)	Hypothetical protein
PA1793	*ppiB*	-3.65	-1.76	Heat-shock[d]	Peptidyl-prolyl cis-trans isomerase B
PA1804	*hupB*	-3.19	-2.54		DNA-binding protei HU
PA1852		-3.42	-3.06	SP (2)	Hypothetical protein
PA1869		-13.92	-3.69	HSL QS (4), SP (2)	Probably acyl carrier protein
PA1887		-3.92	-1.62		Hypothetical protein
PA1888		-5.32	-1.10	SP (2)	Hypothetical protein
PA1939		-3.74	-2.00		Hypothetical protein
PA2037		-3.19	-1.70		Hypothetical protein
PA2223		-4.51	-2.64		Hypothetical protein
PA2300	*chiC*	-7.07	-3.31	HSL QS (4), SP (2)	Chitinase
PA2384		-49.88	-38.37	Fe (6)	Hypothetical protein
PA2386	*pvdA*	-16.14	-23.73	Fe (6), VF[d]	L-ornithine N5-oxygenase
PA2394	*pvdN*	-3.06	-6.32	VF[d]	PvdN
PA2396	*pvdF*	-10.00	-9.76	VF[d]	Pyoverdine synthetase F
PA2397	*pvdE*	-7.31	-13.49	VF[d]	Pyoverdine biosynthesis protein PvdE
PA2398	*fpvA*	-17.44	-20.63	VF[d]	Ferripyoverdine receptor
PA2399	*pvdD*	-3.12	-2.81	VF[d]	Pyoverdine synthetase D
PA2401	*pvdJ*	-3.56	-2.82	VF[d]	PvdJ
PA2411		-3.10	-4.29		Probable thioesterase
PA2412		-4.78	-4.57		Conserved hypothetical protein
PA2426	*pvdS*	-130.15	-115.45	Fe (6), VF[d]	Sigma factor PvdS
PA2570	*pa1L*	-4.38	-1.83	HSL QS (4), SP (2)	LecA
PA2582		-3.24	-2.67		Hypothetical protein
PA2667	*mvaU*	-3.46	-3.94		MvaU
PA2730		-4.93	-5.02		Hypothetical protein
PA2733		-3.26	-2.84		Conserved hypothetical protein
PA2742	*rpmI*	-4.57	-5.56		50S ribosomal protein L35
PA2797		-3.41	-2.19		Hypothetical protein
PA2851	*efp*	-4.20	-2.37		Translation elongation factor P
PA2966	*acpP*	-4.24	-2.11		Acyl carrier protein

PA3049	*rmf*	-9.54	-9.61	Osmotic (7), O_2 (1, 2), SP (2)	Ribosome modulation factor
PA3162	*rpsA*	-3.09	-3.47		30S ribosomal protein S1
PA3190		-3.26	-4.56		Probable binding protein of ABC sugar transporter
PA3341		-6.30	-0.64		Probable transcriptional regulator
PA3385	*amrZ*	-17.48	-1.54	SP (2)	Alginate and motility regulator Z
PA3397	*fprA*	-4.43	-5.03		FprA
PA3440		-3.91	-1.84		Conserved hypothetical protein
PA3450		-4.40	-9.22		Probable antioxidant protein
PA3477	*rhlR*	-6.58	-5.17	SP (2), HSL QS (4), VF[d]	Transcriptional regulator RhlR
PA3496		-4.72	-1.26		Hypothetical protein
PA3520		-3.81	-2.74	SP (2), HSL QS (4)	Hypothetical protein
PA3530	*bfd*	-24.20	-13.65	Fe (6)	Bacterioferritin-associated ferredoxin Bfd
PA3533	*grxD*	-3.22	-1.86		GrxD
PA3621	*fdxA*	-4.19	-1.23		Ferredoxin I
PA3622	*rpoS*	-8.50	-6.24	SP (2)	Sigma factor RpoS
PA3645	*fabZ*	-3.17	-3.77		(3R)-hydroxymyristoyl-[acyl carrier protein] dehydratase
PA3662		-5.12	-0.37	O_2 (1), SP (2)	Hypothetical protein
PA3724	*lasB*	-5.64	-4.57	VF[d], SP (2) HSL QS (4)	Elastase LasB
PA3756		-4.37	-1.78		Hypothetical protein
PA3812	*iscA*	-6.07	-9.35	O_2 (1)	Probable iron-binding protein IscA
PA3813	*iscU*	-7.71	-12.18		Probable iron-binding protein IscU
PA3814	*iscS*	-4.47	-6.75		L-cysteine desulfurase (pyridoxal phosphate-dependent)
PA3815	*iscR*	-5.48	-5.02		IscR
PA3931		-5.89	-13.42		Conserved hypothetical protein
PA3966		-3.34	-2.79		Hypothetical protein
PA4139		-5.20	-3.75		Hypothetical protein
PA4141		-7.91	-8.70	SP (2), HSL QS (4)	Hypothetical protein
PA4156	*fvbA*	-3.00	-1.24		FvbA
PA4211	*phzB1*	-19.06	-12.44	VF[d], SP (2), phenazine[d]	Probable phenazine biosynthesis protein
PA4221	*fptA*	-15.17	-8.18	Fe (6), VF[d]	Fe(III)-pyochelin outer membrane receptor precursor
PA4223		-4.06	-2.25	Fe (6), VF[d]	Probable ATP-binding component of ABC transporter
PA4224	*phcG*	-4.67	-1.86	Fe (6), VF[d]	Pyochelin biosynthetic protein PchG
PA4226	*pchE*	-3.69	-1.41	Fe (6), VF[d]	Dihydroaeruginoic acid synthetase
PA4228	*pchD*	-7.52	-3.11	Fe (6), VF[d]	Pyochelin biosynthesis protein PchD
PA4229	*pchC*	-3.14	-2.30	Fe (6), VF[d]	Pyochelin biosynthetic protein PchC
PA4230	*pchB*	-4.22	-1.56	Fe (6), VF[d]	Salicylate biosynthesis protein PchB
PA4231	*pchA*	-4.20	-1.73	Fe (6), VF[d]	Salicylate biosynthesis isochorismate synthase
PA4232	*ssb*	-3.35	-1.65		Single-stranded DNA-binding protein
PA4238	*rpoA*	-8.94	-10.89		DNA-directed RNA polymerase alpha chain
PA4241	*rpsM*	-4.97	-4.12		30S ribosomal protein S13

PA4242	*rpmJ*	-20.88	-21.40		50S ribosomal protein L36
PA4245	*rpmD*	-4.00	-4.39		50S ribosomal protein L30
PA4246	*rpsE*	-5.65	-5.56		30S ribosomal protein S5
PA4248	*rplF*	-3.55	-2.48		50S ribosomal protein L6
PA4249	*rpsH*	-5.78	-3.81		30S ribosomal protein S8
PA4250	*rpsN*	-4.57	-3.98		30S ribosomal protein S14
PA4251	*rplE*	-5.43	-5.34		50S ribosomal protein L5
PA4253	*rplN*	-5.19	-5.12		50S ribosomal protein L14
PA4254	*rpsQ*	-8.35	-8.65		30S ribosomal protein S17
PA4255	*rpmC*	-9.23	-9.51		50S ribosomal protein L29
PA4256	*rplP*	-4.60	-4.50		50S ribosomal protein L16
PA4257	*rpsC*	-9.48	-9.52		30S ribosomal protein S3
PA4258	*rplV*	-6.20	-5.91		50S ribosomal protein L22
PA4259	*rpsS*	-5.30	-5.35		30S ribosomal protein S19
PA4260	*rplB*	-4.54	-4.58	Nitrosative stress (8)	50S ribosomal protein L2
PA4261	*rplW*	-8.78	-6.48		50S ribosomal protein L23
PA4262	*rplD*	-4.38	-4.15		50S ribosomal protein L4
PA4264	*rpsJ*	-4.53	-3.02		30S ribosomal protein S10
PA4266	*fusA1*	-5.69	-4.82		Elongation factor G
PA4268	*rpsL*	-3.09	-2.64		30S ribosomal protein S12
PA4271	*rplL*	-4.23	-3.48		50S ribosomal protein L7/L12
PA4272	*rplJ*	-13.64	-11.90		50S ribosomal protein L10
PA4273	*rplA*	-5.94	-4.60		50S ribosomal protein L1
PA4274	*rplK*	-12.49	-12.34		50S ribosomal protein L11
PA4276	*secE*	-3.62	-3.11		Secretion protein SecE
PA4296	*pprB*	-3.22	-1.03	SP (2)	Two-component response regulator PprB
PA4370	*icmP*	-4.82	-4.76	Fe (6)	Insulin-cleaving metalloproteinase outer membrane protein precursor
PA4433	*rplM*	-3.47	-1.31		50S ribosomal protein L13
PA4443	*cysD*	-4.82	-6.09		ATP sulfurylase small subunit
PA4451		-4.69	-2.21		Conserved hypothetical protein
PA4468	*sodM*	-8.88	-7.74	Fe (6), H_2O_2 (3)	Superoxide dismutase
PA4469		-30.88	-36.81	Fe (6), SP (2)	Hypothetical protein
PA4470	*fumC1*	-15.70	-13.94	H_2O_2 (3), VF[d]	Fumurate hydratase
PA4471		-83.87	-78.93	Fe (6)	Hypothetical protein
PA4515		-5.27	-5.57	Fe (6)	Conserved hypothetical protein
PA4537		-3.04	-2.49		Hypothetical protein
PA4556	*pilE*	-3.65	-2.67	VF[d]	Type 4 fimbrial biogenesis protein PilE
PA4563	*rpsT*	-7.57	-2.74		30S ribosomal protein S20
PA4568	*rplU*	-3.55	-2.66		50S ribosomal protein L21
PA4570		-65.86	-46.45		Hypothetical protein
PA4638		-29.13	-24.38		Hypothetical protein
PA4723	*dksA*	-3.11	-1.49		Supressor protein DksA
PA4738		-6.10	-3.56	O_2 (2), SP (2)	Conserved hypothetical protein

PA4739		-6.33	-3.25	SP (2), O_2 (2)	Conserved hypothetical protein
PA4843	*gcbA*	-4.62	-1.03	SP (2), cdiGMP (9)	GcbA
PA4853	*fis*	-6.63	-4.00		DNA-binding protein Fis
PA4944	*hfq*	-3.25	-1.97		Hfq
PA5041	*pilP*	-3.04	-2.12	VF[d]	Type 4 fimbrial biogenesis protein PilP
PA5163	*rmlA*	-4.87	-5.48		Glucose-1-phosphate thymidyltransferase
PA5315	*rpmG*	-3.99	-2.46		50S ribosomal protein L33
PA5316	*rpmB*	-3.11	-2.26		50S ribosomal protein L28
PA5348		-3.10	-1.96		Probable DNA-binding protein
PA5351	*rubA1*	-4.35	-1.56		Rubredoxin 1
PA5460	*phoB*	-5.74	-3.89		Two-component response regulator PhoB
PA5531	*tonB*	-4.59	-4.15	Fe (6)	TonB1
PA5557	*atpH*	-3.63	-2.56		ATP synthase delta chain
PA5560	*atpB*	-3.16	-2.46		ATP synthase A chain
PA5563	*soj*	-3.03	-2.95	Nitrosative stress (8)	Chromosome partitioning protein Soj
PA5570	*rpmH*	-3.18	-2.77		50S ribosomal protein L34

Table S2. List of genes with an >3-fold change in gene expression in alginate-encapsulated *P. aeruginosa* when compared to 24 hours planktonic culture of *P. aeruginosa* and the corresponding 24 hours alginate-encapsulated *P. aeruginosa* supplemented with 100 mM KNO_3 as compared to 24 hours planktonic culture.

[a] ID: identification

[b] Detection of >3-fold increase in gene expression of alginate-encapsulated *P. aeruginosa* (Beads) compared to planktonic cells (Planktonic) and the corresponding gene expression when alginate-encapsulated *P. aeruginosa* is compared to alginate-encapsulated *P. aeruginosa* supplemented with nitrate (Beads-NO_3). Bead data is based on 4 biological replicas. Planktonic and Beads-NO_3 data is based on 3 biological replicas.

[c] O_2, oxygen limitation; SP, stationary phase; H_2O_2, peroxide stress; VF, virulence factor; Fe, iron limitation; Mg, magnesium limitation; HSL QS, homoserine lactone based quorum sensing; cdiGMP, cyclic diguanylate GMP.

[d] As annotated at pseudomonas.com (10).

[e] The 141 genes shared between Beads/Planktonic and Beads/Beads-NO_3^- are underlined. All 17 genes from the Beads/Planktonic comparison are shared with Beads/Beads-NO_3^-*, whereas 87 of the 153 downregulated genes have a similar >3-fold differential expression.*

[f] The 13 genes shared between Beads/Planktonic and Beads-NO_3^-/Planktonic are all downregulated, and are marked in *italic* in Table S2.

[g] The two genes which share a similar >3-fold change between all three comparisons (beads/planktonic, beads/beads-NO_3^- and beads-NO_3^-/planktonic) are highlighted in gray. The same two genes are shared between Beads/Beads-NO_3^- and Beads-NO_3^-/Planktonic, according to Venn-diagramm (Figure 8A).

All genes encoding ribosomal proteins have been boxed.

1. Trunk K*, et al.* (2010) Anaerobic adaptation in Pseudomonas aeruginosa: definition of the Anr and Dnr regulons. *Environmental microbiology* 12(6):1719-1733.
2. Alvarez-Ortega C & Harwood CS (2007) Responses of Pseudomonas aeruginosa to low oxygen indicate that growth in the cystic fibrosis lung is by aerobic respiration. *Molecular microbiology* 65(1):153-165.
3. Chang W, Small DA, Toghrol F, & Bentley WE (2005) Microarray analysis of Pseudomonas aeruginosa reveals induction of pyocin genes in response to hydrogen peroxide. *BMC genomics* 6:115.
4. Hentzer M*, et al.* (2003) Attenuation of Pseudomonas aeruginosa virulence by quorum sensing inhibitors. *The EMBO journal* 22(15):3803-3815.
5. McPhee JB*, et al.* (2006) Contribution of the PhoP-PhoQ and PmrA-PmrB two-component regulatory systems to Mg2+-induced gene regulation in Pseudomonas aeruginosa. *Journal of bacteriology* 188(11):3995-4006.

6. Ochsner UA, Wilderman PJ, Vasil AI, & Vasil ML (2002) GeneChip expression analysis of the iron starvation response in Pseudomonas aeruginosa: identification of novel pyoverdine biosynthesis genes. *Molecular microbiology* 45(5):1277-1287.
7. Aspedon A, Palmer K, & Whiteley M (2006) Microarray analysis of the osmotic stress response in Pseudomonas aeruginosa. *Journal of bacteriology* 188(7):2721-2725.
8. Firoved AM, Wood SR, Ornatowski W, Deretic V, & Timmins GS (2004) Microarray analysis and functional characterization of the nitrosative stress response in nonmucoid and mucoid Pseudomonas aeruginosa. *Journal of bacteriology* 186(12):4046-4050.
9. Kulasakara H*, et al.* (2006) Analysis of Pseudomonas aeruginosa diguanylate cyclases and phosphodiesterases reveals a role for bis-(3'-5')-cyclic-GMP in virulence. *Proceedings of the National Academy of Sciences of the United States of America* 103(8):2839-2844.
10. Winsor GL*, et al.* (2016) Enhanced annotations and features for comparing thousands of Pseudomonas genomes in the Pseudomonas genome database. *Nucleic acids research* 44(D1):D646-653.
